# Network phenotypes and their clinical significance in temporal lobe epilepsy using machine learning applications to morphological and functional graph theory metrics

**DOI:** 10.1038/s41598-022-18495-z

**Published:** 2022-08-24

**Authors:** Camille Garcia-Ramos, Veena Nair, Rama Maganti, Jedidiah Mathis, Lisa L. Conant, Vivek Prabhakaran, Jeffrey R. Binder, Beth Meyerand, Bruce Hermann, Aaron F. Struck

**Affiliations:** 1 Department of Medical Physics, University of Wisconsin-Madison, Madison, USA; 2 Department of Neurology, University of Wisconsin-Madison, Madison, USA; 3 Department of Radiology, University of Wisconsin-Madison, Madison, USA; 4 Department of Neurology, Medical College of Wisconsin, Milwaukee, USA; 5 William S Middleton VA Hospital, Madison, WI USA

**Keywords:** Paediatric research, Cognitive neuroscience

## Abstract

Machine learning analyses were performed on graph theory (GT) metrics extracted from brain functional and morphological data from temporal lobe epilepsy (TLE) patients in order to identify intrinsic network phenotypes and characterize their clinical significance. Participants were 97 TLE and 36 healthy controls from the Epilepsy Connectome Project. Each imaging modality (i.e., Resting-state functional Magnetic Resonance Imaging (RS-fMRI), and structural MRI) rendered 2 clusters: one comparable to controls and one deviating from controls. Participants were minimally overlapping across the identified clusters, suggesting that an abnormal functional GT phenotype did not necessarily mean an abnormal morphological GT phenotype for the same subject. Morphological clusters were associated with a significant difference in the estimated lifetime number of generalized tonic–clonic seizures and functional cluster membership was associated with age. Furthermore, controls exhibited significant correlations between functional GT metrics and cognition, while for TLE participants morphological GT metrics were linked to cognition, suggesting a dissociation between higher cognitive abilities and GT-derived network measures. Overall, these findings demonstrate the existence of clinically meaningful minimally overlapping phenotypes of morphological and functional GT networks. Functional network properties may underlie variance in cognition in healthy brains, but in the pathological state of epilepsy the cognitive limits might be primarily related to structural cerebral network properties.

## Introduction

In temporal lobe epilepsy (TLE), abnormalities in brain structure^1–3^, connectivity^4,5^, and cognition^6,7^ are consistently demonstrated. The abnormalities range from reduced gray and white matter volumes and thickness to abnormal diffusion and differences in functional connectivity patterns. While these findings are reproducible it is also evident that there is meaningful heterogeneity across patients, even within specific syndromes of TLE such as mesial temporal lobe epilepsy^5^. This variability challenges whether TLE should be studied as a single population. The question that has arisen is whether there are specific subgroups of TLE patients with unique patterns of structural or functional abnormality, specifically, whether there are clusters of patients with similar findings who are distinct from other subgroups of TLE patients.

The presence of latent groups or phenotypes has been shown to be the case in relation to cognition in TLE^8,9^ with 3 to 4 cognitive subtypes including a group that is comparable to controls (i.e., showing no cognitive impairment), a group with atypical (for a focal epilepsy) generalized cognitive impairment, and 1 to 2 groups with focal language, memory, and/or executive dysfunction (see^10^ for review). TLE phenotypes have also been identified regarding mesiotemporal structural abnormalities including hippocampus, entorhinal cortex, and amygdala^11^. One of the questions addressed here is whether subgroups or phenotypes of morphological and functional network integrity can be identified, and their significance determined through their associations with demographic, clinical and cognitive variables. Here we address this issue by applying K-means clustering to morphologically (cortical and subcortical brain volumes) and functionally derived (resting-state functional Magnetic Resonance Imaging (RS-fMRI)) GT metrics from individual TLE subjects. K-means clustering is an unsupervised machine learning algorithm that forms clusters within the studied group by minimizing the distance between GT metrics^12–14^. The optimal number of clusters was determined using the elbow method^15^ and Silhouette method^16^. In the case of this study, three GT measures: global efficiency, local efficiency, and modularity index (Q) were used to form clusters. Graph theory (GT) is a methodology that allows the investigation of the brain networks by treating brain regions as nodes with connections or edges being determined by measures of associations between regions (e.g., Blood Oxygenation Level Dependent (BOLD) response, diffusion weighted imaging measures, volumetric correlations)^17,18^. It is a methodology capable of interrogating the brain regarding its global network properties. K-means clustering has been previously applied to brain function (i.e., rs-fMRI) in order to determine, for example, dynamic functional network connectivity states of TLE patients^9^ on networks derived from Independent Component Analysis (i.e., using more conventional uses of the data). To our knowledge, this methodology has not been applied to GT metrics in TLE. Given that GT provides assessment of the brain at the global and local levels, as well as in terms of its configuration, we hypothesize that its use will capture subtleties within the TLE group that will make possible cluster identification with clinical and cognitive associations.

Another question that we address here is to test the association between functional, structural, and cognitive phenotypes in TLE. There is vast evidence regarding functional discrepancies in TLE compared to controls, in terms of BOLD response (i.e., brain activation /deactivation), functional connectivity, and even GT analyses^19–21^. Furthermore, morphological measures, like cortical volumes and diffusion weighted images, have shown differences in TLE compared to controls^22,23^. Even though functional and morphological analyses do not always coincide in terms of the disrupted areas in TLE, we hypothesize that associations between their found phenotypes will be observed.

## Results

Compared to controls (n = 36) the participants with TLE (n = 97) were significantly older (t(131) = 2.589, p = 0.011), had a lower full-scale IQ (Intelligence Quotient) (t(131) = − 5.431, p < 0.001), and fewer years of education (t(131) = − 3.214, p = 0.002); but were similar in gender distribution ($\chi$^2^ = 3.25, p = 0.06). Given that the cognitive tests in this study were adjusted for age, no additional age-related adjustments for cognitive analyses were made.

### Functional and morphological K-means clustering and their association with cognitive phenotypes

**Figure 1:**
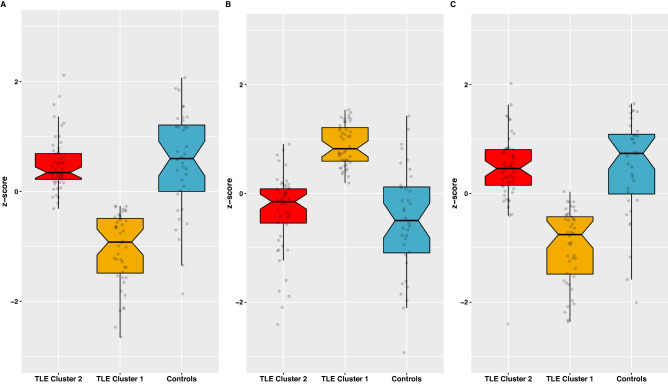
(**A**) Local efficiency, (**B**) Global efficiency, and (**C**) modularity index across ML-derived TLE clusters (red and yellow) and healthy controls (blue) for RS-fMRI.

**Figure 2:**
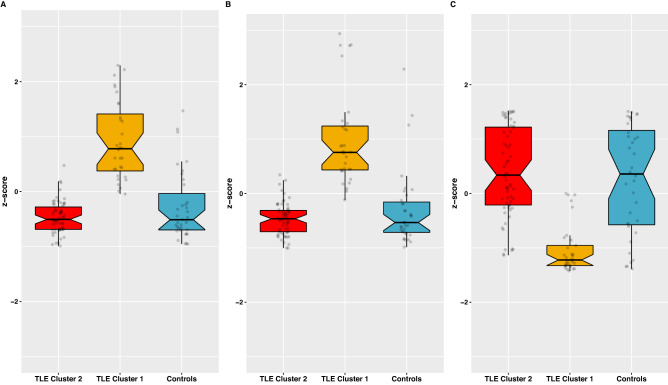
(**A**) Local efficiency, (**B**) Global efficiency, and (**C**) modularity index across ML-derived TLE clusters (red and yellow) and healthy controls (blue) for morphological MRI.

**Figure 3:**
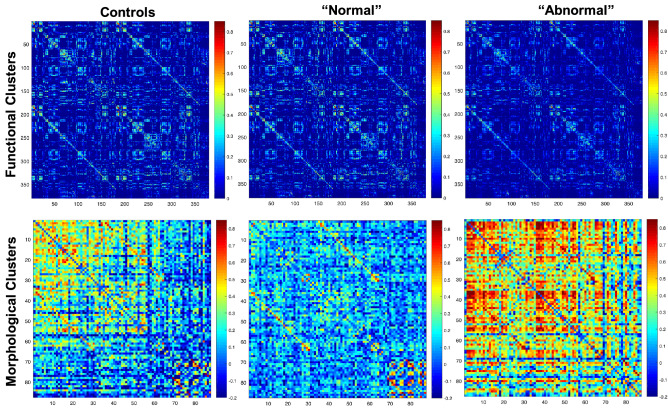
Adjacency matrices of functional (top) and morphological (bottom) correlation matrices on controls (left), “normal” clusters (middle), and “abnormal clusters” (right).

Global efficiency, mean local efficiency, and modularity index (Q) were the GT measures used in the K-means clustering algorithm for functional and then morphological analyses. In each case, two clusters were found within the TLE group: one like controls (*Normal*), and one significantly different from controls (*Abnormal*) (Figs. 1 and 2). For the functional analysis, there were 51 (53%) patients in the *Normal* cluster, and 46 (47%) in the *Abnormal* one. For the morphological analysis there were 73 (75%) in the *Normal* cluster and 24 (25%) in the A*bnormal* cluster. Correlation matrices were calculated for each analysis (Fig. 3) which revealed opposite patterns for clusters in terms of functional and morphological GT, where “abnormal” clusters showed higher and widespread correlations for morphological GT while the opposite was true for functional GT.

**Figure 4:**
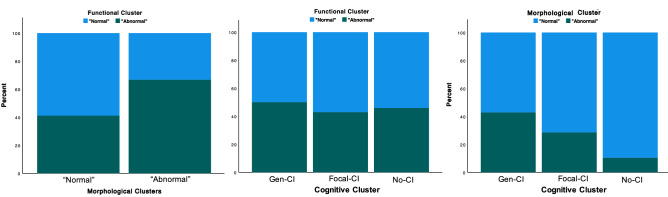
(Left) Proportion of TLE participants in morphological clusters vs functional clusters, (middle) proportion of TLE participants in cognitive clusters vs functional clusters, and (right) proportion of TLE participants in cognitive clusters vs morphological clusters. Results were significant for the *left* and the *right* cases.

The distribution of cognitive phenotypes was examined as a function of the K-means functional and morphological cluster groups (Fig. 4). There was a significant association between the previously established TLE cognitive phenotypes (see Hermann et al.^24^) and morphological clusters ($\chi$^2^ = 4.737, p = 0.026). The more abnormal cognitive phenotypes were associated with the *Abnormal* morphological cluster, the cluster that was most dissimilar to controls. The cognitive phenotypes did not show a significant association with the functional clusters. In terms of overlap between functional and morphological clusters, there was 44% (43 subjects) correspondence for the *Normal* cluster, and 16% (16 subjects) for the *Abnormal* one, therefore, there was only partial overlap of subjects between clusters ($\chi$^2^ = 8.207, p = 0.017). Table 1 shows the composition of clusters along with clinical and demographic variables.

**Table 1:** Functional and morphological clusters.

	Controls (n = 36)	TLE (n = 97)	Functional clusters		Morphological clusters	
			*Normal* (n = 51)	*Abnormal* (n = 46)	*Normal* (n = 73)	*Abnormal* (n = 24)
Age^a,c^ (mean ± SD)	34.1 ± 10.8	40.0 ± 11.8	37.2 ± 11.0	42.7 ± 11.9	39.9 ± 10.9	39.1 ± 14.4
Gender^b^ (M/F)	20/16	37/60	19/32	18/28	21/52	16/8
Education^c^ (mean ± SD)	16.5 ± 2.8	14.8 ± 2.8	14.92 ± 2.6	14.67 ± 3.0	15.1 ± 2.8	13.9 ± 2.7
FSIQ^b,c^ (mean ± SD)	115.3 ± 12.4	100.8 ± 14.1	102.53 ± 12.85	100.33 ± 14.2	103.5 ± 12.6	95.1 ± 14.4
Age at first seizure (mean ± SD)	–	22.2 ± 14.4	21.5 ± 12.9	23.0 ± 16.0	22.0 ± 13.5	22.9 ± 17.2
Number of ASM (mean ± SD)	–	1.78 ± 0.95	1.67 ± 0.93	1.88 ± 0.95	1.71 ± 0.9	1.95 ± 1.1
Medication refractory (0/1)	–	36/61	21/30	15/31	28/45	8/16
Seizure lateralization (L/R/B/U)	–	17/48/22/7	27/10/4/10	21/12/3/7	38/14/7/13	10/8/0/4
Number of lifetime GTC^b^ (mean ± SD)	–	12.4 ± 22.5	10.0 ± 15.7	15.4 ± 28.7	8.8 ± 17.4	25.0 ± 32.4
WASI vocabulary^b,c^ (mean ± SD)	61.6 ± 9.2	50.9 ± 8.8	51.6 ± 9.1	50.1 ± 8.5	52.4 ± 8.6	46.2 ± 8.0
WASI block design^c^ (mean ± SD)	56.17 ± 9.5	50.0 ± 10.1	51.4 ± 8.9	48.6 ± 11.1	51.1 ± 9.4	46.8 ± 11.3
Boston naming test^c^ (mean ± SD)	48.5 ± 10.4	43.3 ± 11.3	42.9 ± 11.2	43.7 ± 11.4	42.6 ± 10.7	45.3 ± 12.8
Grooved pegboard-dominant hand^b,c^ (mean ± SD)	9.9 ± 3.1	7.9 ± 2.9	8.3 ± 3.0	7.5 ± 2.8	8.5 ± 2.9	6.1 ± 2.2
Grooved pegboard-nondominant hand^b,c^ (mean ± SD)	10.8 ± 1.8	8.3 ± 2.7	8.7 ± 2.8	7.9 ± 2.6	8.8. ± 2.6	6.8 ± 2.6
Rey auditory verbal learning test^c^ (mean ± SD)	104.6 ± 11.6	91.7 ± 16.3	92.1 ± 16.9	91.1 ± 15.8	93.1 ± 16.5	87.4 ± 15.3

*L* left, *R* right, *B* bilateral, *U* unknown.

^a^Significantly different between functional clusters.

^b^Significantly different between morphological clusters.

^c^Significantly different between controls and TLE.

### Association of functional and morphological clusters with demographic and clinical variables

**Figure 5:**
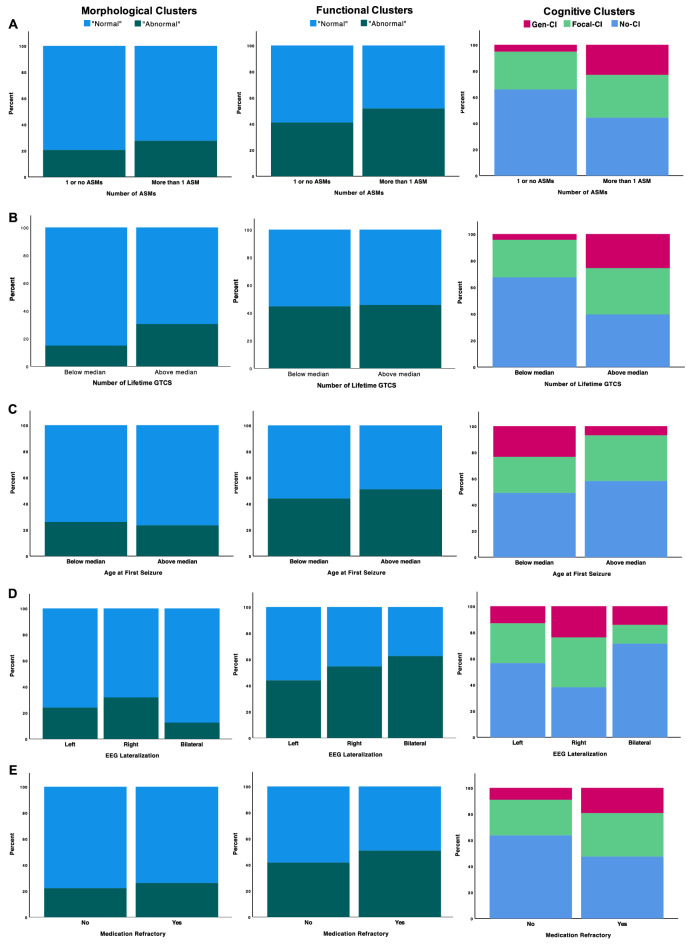
Proportion of TLE participants in (left) morphological clusters, (middle) functional clusters, and (right) cognitive clusters vs (**A**) number of ASMs (binarized), (**B**) lifetime number of GTCs (binarized), (**C**) age at first seizure (binarized), (**D**) EEG lateralization, and (**E**) medication refractory.

The relationship between the functional and morphological clusters with categorical clinical epilepsy variables was undertaken [medication refractory (yes/no), number of antiseizure medications (ASMs) (binarized), seizure lateralization (left, right, bilateral, unknown), age at first seizure (binarized), and estimated lifetime number of generalized tonic–clonic seizures (GTCS) (binary)]. Figure 5 shows the proportions of each TLE cluster (i.e., morphological, functional, cognitive) as a function of the categorical clinical variables. The number of ASMs and lifetime number of GTCS were significant. For ASM, only cognitive clusters were statistically significant ($\chi$^2^ = 6.491, p = 0.039). For lifetime number of GTCS, morphological clusters are trending toward significance ($\chi$^2^ = 3.212, p = 0.061), and cognitive clusters were statistically significant ($\chi$^2^ = 10.368, p = 0.006).

Age was significantly different between functional clusters (t(95) = − 2.47, p = 0.015), while gender ($\chi$^2^ = 11.0, p = 0.001), FSIQ (t(95) = 2.836, p = 0.006), and lifetime number of GTCS (t(91) = − 3.018, p = 0.003) were significantly different between morphological clusters (see Table 1). The remaining variables (i.e., age at first seizure, number of ASM, medication refractory, and seizure lateralization) did not show significant differences between clusters for either analysis.

### Association of individual cognitive measures with GT metrics

Lastly, in both the control and TLE groups associations between global GT metrics from the functional and morphological analyses and targeted individual cognitive measures were examined via Spearman correlations. These correlations were performed to directly compare associations within the controls and TLE groups to determine symmetry/asymmetry of relationships between cognition and morphological and functional metrics. As shown in Table 2, among the controls, functional GT measures had significant associations with vocabulary/word knowledge, visual object naming, and speeded fine motor dexterity; with no relationship with visuoconstruction or verbal learning/memory. In contrast, for TLE participants morphological GT metrics were significantly associated with all cognitive abilities with functional GT metrics also associated only with motor speed/dexterity.

**Table 2:** Significant correlations between GT measures and cognition.

	Control	TLE
WASI vocabulary subtest	fGE ($R=-0.351, p=0.036$) fLE ($R=0.384, p=0.021$)	mQ ($R=0.227, p=0.026$) mGE ($R=-0.336, p=0.001$) mLE ($R=-0.301, p=0.003$)
WASI block design subtest		mQ ($R=0.21, p=0.039$) mGE ($R=-0.228, p=0.025$) mLE ($R=-0.248, p=0.014$)
Boston naming test	fQ ($R=0.371, p=0.026$)	
Grooved pegboard-dominant hand	fQ ($R=0.343, p=0.04$) fLE ($R=0.353, p=0.035$)	mQ ($R=0.315, p=0.002$) mGE ($R=-0.441, p<0.001$) mLE ($R=-0.428, p<0.001$) fGE ($R=-0.236, p=0.02$)
Grooved pegboard-nondominant hand	fQ ($R=0.424, p=0.01$) fGE ($R=-0.43, p=0.009$) fLE ($R=0.468, p=0.004$)	mQ ($R=0.21, p=0.039$) mGE ($R=-0.356, p<0.001$) mLE ($R=-0.35, p<0.001$) fGE ($R=-0.228, p=0.025$) fLE ($R=0.221, p=0.039$)
Rey auditory verbal learning test (total words recalled)		mGE ($R=-0.242, p=0.017$) mLE ($R=-0.221, p=0.029$)

*f* functional, *m* morphological, *GE* global efficiency, *LE* local efficiency.

## Discussion

In this investigation, machine learning (K-means clustering) was employed on two distinct sets of global graph theory metrics from TLE participants: one focused on brain morphology (i.e., cortical and subcortical volumes), and the other focused on brain function (i.e., RS-fMRI). Three main findings resulted: (1) K-means clustering revealed two distinct phenotypes of morphological as well as functional network status in patients with TLE—*Normal* (functional: 53%, morphological: 75%) and *Abnormal* (functional: 47%, morphological: 25%). (2) These clusters only partially overlapped indicating substantial independence of functional and morphological network abnormalities in persons with TLE. (3) Cluster membership was of clinical significance as demonstrated by their association with multiple clinical seizure features and cognition.

This is the first investigation to use GT to analyze both morphological and functional imaging data as well as to demonstrate the existence of discrete network phenotypes in this fashion. Multiple papers have reported abnormalities in morphological and functional networks in TLE patients (for a review, see Bernhardt et al.^25^). However, in this investigation we were able to demonstrate separate latent groups within each analysis. Interestingly, a substantial proportion of TLE participants were comparable to controls, this trend being more pronounced in the morphological analysis. These findings agree with other cognitive and behavioral studies where cluster analysis work has shown a significant proportion of patients with TLE to exhibit cognitive and behavioral performance comparable to controls, with other groups showing abnormalities in type and/or magnitude^9,24^. In this case, the abnormal functional cluster had higher global efficiency and lower local efficiency and Q, while the abnormal morphological cluster had higher global and local efficiency but lower Q than controls. The direction of local efficiency was opposite between morphological and functional clusters showing that tightly connected small networks are associated with pathology in the morphologic grouping, but it is the breakdown of these local connections that are associated with pathology in functional networks, a finding evident qualitatively from the adjacency matrices (Fig. 3). The increased morphological local efficiency may be related to patterns of atrophy along the epileptic network that reflect disease severity. The opposite finding in functional connectivity may be related to a reorganization of connectivity secondary to the disruption from the primary epileptic network. Importantly, a considerable proportion of TLE participants did not exhibit these changes and were comparable to controls, or *normal,* is important and points to the spectrum of abnormality that should be understood and captured in these investigations, the etiology underlying this spectrum remaining to be investigated, and their implications understood. In addition, participants within the functional and morphological clusters did not overlap significantly, meaning that patients with *abnormal* brain function did not necessarily present *abnormal* brain morphology, and vice versa. This apparent dissociation between function and structure in TLE, although seemingly unanticipated, has been observed before^26^ and suggests that future multimodal imaging investigations with additional imaging measures may reveal interesting associations and dissociations.

We demonstrated that functional and morphological clusters had some overlapping but many unique associations with clinical and demographic variables (Table 1, Fig. 4). Morphological clusters appeared more sensitive to demographic, clinical and cognitive variables, that is, they were significantly different in gender, the number of estimated number of lifetime GTCS, cognitive phenotypes and several individual cognitive tests such as FSIQ. In contrast, functional clusters differed only in age. Therefore, abnormalities in brain morphology and function in TLE might be linked to different factors that, in this case, do not overlap significantly. There have been investigations using structural brain data to predict postoperative seizure outcomes of patients with TLE^27,28^. For example, Bonilha et al.^27^ used structural GT to predict seizure outcomes in TLE after temporal lobectomy and found that network characteristics of white matter integrity along with clinical variables served as outcome predictors. Furthermore, Doucet et al.^28^ studied gray matter integrity of the frontal lobe of pre-surgical TLE patients using voxel-based morphometry (VBM) and were able to predict seizure outcomes in some patients. Given that the current investigation was able to differentiate TLE participants into *normal* and *abnormal* phenotypes, this might help yield greater precision in predicting clinical and surgical outcomes.

Evidence that the functional and morphological clusters have potential for clinical utility is their association with clinical measures such as the estimated frequency of lifetime generalized seizures as well as cognitive measures. A reliable finding in the neuropsychology of epilepsy is the existence of discrete cognitive subgroups ranging from intact cognition comparable to controls, to expected focal cognitive abnormalities, to widespread and arguably unexpected (for a focal epilepsy) generalized cognitive impairment^9,24,29^. The degree to which disordered morphological versus functional networks may contribute to the existence of these groups, as well as to performance on individual cognitive tests, is a topic of contemporary interest. Among TLE patients, it was clear that the abnormal morphological network phenotype was more closely associated with cognitive phenotypes than the disordered functional network phenotype (Fig. 4). To determine whether there was a different pattern between controls and TLE patients we also investigated individual cognitive tests from the domains of intelligence, verbal learning and memory, naming, and speeded motor dexterity; and again, it was clear that performance in TLE was linked to morphological network measures while for controls functional metrics predominated. Such TLE-specific morphological associations with cognition might deem morphological GT measures more susceptible to disruptions than functional GT measures.

A prior investigation of resting-state functional connectivity in the Epilepsy Connectome Project (ECP) data set demonstrated differences between TLE and controls in global connectivity using the GT measures of global clustering coefficient and rich club proportion^21^. These findings also correlated with neuropsychological disease severity. Thus, while the abnormal morphological phenotype and its associated characteristics are more tightly associated with cognition within the current experiment, other GT methods of quantifying aberrant global connectivity can detect differences, highlighting the sensitivity not only to modality (morphology versus resting-state functional connectivity), but also to the evaluation metrics. Rich club proportion and global clustering coefficient measure “clustering” tendencies of nodes, especially highly connected nodes in the case of rich club proportion. Whereas global efficiency and local efficiency are related to path length between nodes, and Q detects a tendency for forming networks but on a larger scale than “clustering” measures, with less focus on highly connected networks like rich club proportion. The current study is an extension of prior investigations using efficiency and modularity measures in morphological connectivity^30,31^. It was logical to treat the morphological and functional measures as similar as possible therefore, efficiency and modularity measures were selected. Nonetheless this study highlights the importance of feature selection in machine-learning.

The use of ML on GT metrics based on functional and morphological data, compared to more traditional analyses (i.e., seed based functional connectivity, cortical and subcortical volumes/thickness), facilitated the identification of novel discrete latent functional and morphological groups with clinical and cognitive correlates. Abnormalities in GT metrics of functional connectivity are more related to cognition in controls and morphology more relevant in epilepsy. The findings suggest that at the global network level the functional connectivity may underlie variance in cognition in healthy brains, but in the pathological state of epilepsy the cognitive limits might be primarily related to structural network changes. It may be that in healthy controls the variance in morphology is smaller than in the pathological state and thus it follows that associations with cognition would be greater with functional connectivity. In contradistinction is TLE where patterns of cortical thinning and atrophy are intrinsic to the disease and more tightly associated with cognition and disease specific factors like lifetime number of GTCs. The functional connectivity in these patients, at least as measured with the three global GT measures presented here, were not as effective in capturing the relevant clinical factors like seizure burden or cognitive decline. Potentially looking at other global GT metrics (such as Rich Club Proportion and Global Clustering Coefficient) could be more relevant—highlighting how functional and morphological connectivity are measuring fundamentally different elements of pathophysiology. This orthogonality should be exploited in future phenotyping efforts or in developing combinatoric biomarkers. The essential finding is that morphologic and functional connectivity provide independent information.

Even though the data from this investigation were carefully obtained, this manuscript has some limitations. One pertains to the relatively modest sample size of the control group. Although the analysis was mainly focused on TLE participants, a greater number of controls would have been favorable for demographic and cognitive comparisons. Another limitation is the age discrepancy between controls and TLE, and between some of the obtained clusters. Given that the cognitive measures used in this analysis were already age-corrected, the results should not represent age-related differences, however, it is a matter that should be acknowledged. We want to emphasize that morphological matrices were calculated indirectly with a statistical methodology proposed by Saggar et al.^32^. Although it was proven to reflect structural connectivity networks such matrices are based on the brain volumes correlations but are not volumetric correlations. Lastly, as in all clustering techniques the clustering algorithm is sensitive to the type of data the algorithm is clustering and the underlying clinical heterogeneity. All reasonable attempts to ensure that the clusters are reflective of distinct groups through data-driven clustering techniques were performed. Future studies should be aimed at confirming these clusters are similar in independent populations, incorporating other types of data (e.g. EEG), and correlate with pre-clinical and long-term clinical trajectory.

## Methods

### Participants

Participants include 97 TLE patients and 36 healthy controls from the ECP^33,34^ (Table 1), a project that our group is a part of. ECP is a two-site research project involving the Medical College of Wisconsin and the University of Wisconsin-Madison. The dataset generated during the current study is not publicly available but it is projected to be released in Summer 2022. However, data are available from the corresponding author on reasonable request. TLE participants were between the ages of 19 to 60 with tested FSIQ at or above 70, speak English fluently, with no medical contraindications to MRI. Some TLE participants (63%) had medically refractory epilepsy defined as a disabling seizure during the last year despite having tried at least 2 appropriate anti-seizure medications (ASM). The diagnosis of TLE and side of seizure onset were determined by a board‐certified neurologist with expertise in epileptology, in accordance with the criteria defined by the International League Against Epilepsy, based on scalp or intracranial video‐electroencephalographic (EEG) telemetry, seizure semiology, and neuroimaging evaluation. Specifically, TLE participants met two or more of the following criteria: (1) described or observed clinical semiology consistent with seizures of temporal lobe origin, (2) EEG evidence of either Temporal Intermittent Rhythmic Delta Activity or temporal lobe epileptiform discharges, (3) temporal lobe onset of seizures captured on video EEG monitoring, or (4) MRI evidence of mesial temporal sclerosis or hippocampal atrophy. Patients with any of the following were excluded: (1) lesions other than mesial temporal sclerosis causative for seizures, and (2) an active infectious/autoimmune/inflammatory etiology of seizures.

Controls were healthy adults between the ages of 18 and 60. Exclusion criteria included: Edinburgh Laterality (Handedness) Quotient less than +50; primary language other than English; history of any learning disability, brain injury or illness, substance abuse, or major psychiatric illness (major depression, bipolar disorder, or schizophrenia); current use of vasoactive medications; and medical contraindications to MRI. All participants provided written informed consent, and the study was reviewed and approved by the IRB (Institutional Review Board) at Medical College of Wisconsin and all experiments were performed in accordance with relevant guidelines and regulations.

### Neuroimaging acquisition

MRI was performed on 3 T General Electric (GE) 750 scanners at both institutions. T1-weighted structural images were acquired using MPRAGE (reduced magnetization prepared gradient echo sequence): TR/TE = 604 ms/2.516 ms, TI = 1060.0 ms, flip angle = 8°, FOV = 25.6 cm, voxel size = 0.8 mm isotropic. Four 5-min rs-fMRI images were acquired over two sessions using whole-brain simultaneous multi-slice, gradient echo planer imaging^35^: 8 bands (72 slices, TR/TE = 802 ms/33.5 ms, flip angle = 50°, matrix = 104 × 104, FOV = 20.8 cm, voxel size = 2.0 mm isotropic) and a 32-channel receive coil were concatenated. The participants were asked to fixate on a white cross at the center of a black screen during the scans for better reliability^36^.

### Neuropsychological assessment

The healthy control and epilepsy participants underwent neuropsychological evaluation that included measures of intelligence (Wechsler Abbreviated Scale of Intelligence-2 Vocabulary and Block Design subtests)^37^, verbal learning and memory (Rey Auditory Verbal Learning Test) including total words learned across trials and delayed recall^38^, object naming (Boston Naming Test)^39^, letter fluency (Controlled Oral Word Association Test)^40,41^, semantic fluency (Animal Naming)^40,42^, spatial orientation (Judgement of Line Orientation)^43^, face recognition (Facial Recognition Test)^43^, and speeded fine motor dexterity (Grooved pegboard, dominant and non-dominant hands)^44^. In addition, selected cognitive subtests from the National Institutes of Health Toolbox-Cognitive Battery were included: l Pattern Comparison Processing Speed^45,46^, Dimensional Change Card Sort, List Sorting Working Memory, Flanker Inhibitory Control and Attention, Picture Vocabulary, Oral Reading Recognition, and Picture Sequence Memory tests. Legal copyright restrictions prevent public archiving of the various neuropsychological tests used in the study. These can be obtained from the copyright holders in the cited references accompanying each test.

### Cognitive clusters

Morphological and functional clusters were compared to empirically derived neuropsychological cognitive phenotypes identified in this patient cohort (see Hermann et al.^24^). In short, 18 neuropsychological test metrics were assigned to 5 cognitive domains and subsequently subjected to cluster analysis that revealed three cognitive phenotypes including: (a) a cluster with cognitive performance similar to controls (No-Cognitive Impairment (CI), n = 57), (b) a cluster with cognitive abnormality characterized by leading impairment in language, memory and executive function (Focal-CI, n = 34), and (c) a cluster with performance impaired across all the tested cognitive domains including language, memory, executive function, visuospatial and motor speed (Generalized-CI, N = 20) (Fig. 1S).

### MRI preprocessing

MR images were processed using the Human Connectome Project (HCP) minimal processing pipelines^47^ which are primarily based on FreeSurfer v5.3^48^ and FSL (Functional MRI of the brain Software Library)^49^. In brief, the T1-weighted images are non-linearly registered to the MNI (Montreal Neurological Institute) space, segmented into predefined structures, white and pial cortical surfaces reconstructed, followed by standard folding-based surface registration to a surface atlas (the “fsaverage” template). The functional portion of the pipelines removes nonlinear spatial distortions in the rs-fMRI images using spin echo unwarping maps, realigns volumes to compensate for subject motion, registers to the structural images, reduces the bias field, normalizes the 4D image to a global mean, masks the data with the final brain mask and maps the voxels within the cortical gray matter ribbon onto the native cortical surface space. General details on the HCP processing pipelines can be found in Glasser et al.^47^, and specific details in our previous work^34^. The Desikan-Killiany atlas was used for the segmentation of cortical areas while an atlas that contains probabilistic information on the location of structures was used for the segmentation of subcortical regions^52^. Cortical and subcortical volumes were extracted from FreeSurfer in order to construct correlation matrices for the GT analysis.

The functional portion of the pipelines removes spatial distortions in the rs-fMRI images using spin echo unwarping maps, realigns volumes to compensate for subject motion, registers to the structural images, reduces the bias field, normalizes the 4D image to a global mean, masks the data with the final brain mask and maps the voxels within the cortical gray matter ribbon onto the native cortical surface space. Details on the HCP processing pipelines can be found in Glasser et al.^47^. Additional pre-processing was performed on the rs-fMRI images using AFNI (Analysis of Functional Neuro-Images)^50^. This included motion regression using 12 motion parameters, regression-based removal of signal changes in the white matter, CSF, global signal, and band-pass filtering (0.01–0.1 Hz). Time-series data from four 5-min rs-fMRI scans acquired in a single session were concatenated. 360 time-series from Glasser Parcellation^51^ plus 19 FreeSurfer subcortical regions^52^ were extracted per subject. Pairwise Pearson correlations between 379 time series were calculated and Fisher-z transformed for generating connectivity matrices.

### Matrix and graph theory measures: calculations and statistical analyses

Two GT analyses were performed: one based on morphological matrices (i.e., based on cortical and subcortical volumes), and one based on functional matrices (i.e., resting-state fMRI). The morphological analysis comprised 87 nodes containing cortical regions (frontal, temporal, parietal, and occipital areas) based on the Desikan-killiany atlas from FreeSurfer, and subcortical structures. We calculated a weighted undirected matrix based on the correlation coefficients of the covariance between nodal volumes corresponding to each participant. This was done by applying the AOP (add one patient) approach formulated by Saggar et al.^32^. With this method, one of the TLE participants was added to the control group before the correlation matrix was calculated. Then the matrix of the control group was subtracted from the one containing the controls plus one TLE, and a matrix reflecting the effect of that single TLE participant was obtained. This was done for each TLE participant (n = 97). Afterwards, GT metrics were calculated, correcting for intracranial volume (ICV). The GT analysis on functional matrices was based on the Glasser Parcellation^51^, which comprises 379 nodes encompassing both cortical and subcortical regions. Functional matrices based on temporal correlations between nodes were constructed for each subject and graph theory metrics were then calculated. Tables 1S and 2S (supplemental file) contain the list of the 87 nodes used for the morphological analysis, and the 379 nodes used for the functional analysis, respectively.

Once the matrices were created, they were thresholded using a combination of proportional thresholding with the Minimum Spanning Tree (MST) as its backbone (to avoid *disconnected* nodes). In this case, a density of 25% means the MST plus 25% of the rest of the strongest correlations from the matrix (see Garcia‐Ramos et al.^53^ for details). The degree distribution for each imaging modality can be found in Fig. 3S of the supplementary document.

The graph theory measures used in this investigation were global efficiency, local efficiency, and Q because they capture the association between nodes at both global and regional levels^54^, as well as their configuration^55^, respectively. Global efficiency is defined as the average of the inverse of the shortest paths in the network^54^. Therefore, high global efficiency represents the integration of communication within the network. In contrast, local efficiency is a metric of interactions between the neighbors of a node^54^. Specifically, it is a measure based on the shortest paths between each node's neighbors, which reflects how efficient communication is between the immediate spatial neighbors of a node^54^. The modularity index (Q) reflects how well the global network of nodes can be divided into highly connected sub-networks or modules that often contribute to the same underlying neurological processes^55,56^. In a highly modular network, nodes within the same modules are said to be working toward the same process. In this study, Q was estimated using the modularity Louvain algorithm.

Since different thresholds could render different results, we devised a method to identify a threshold that captured the network configuration. Given that modularity assesses network configuration, we calculated it 1000 times for averaged matrices at every threshold. This was done since the Modularity Louvain algorithm estimates modularity instead of performing an exact calculation^55^. Then, we obtained the highest proportion regarding the number of modules at the given threshold and found the one at which the number of modules reached a constant value for each group and for each GT analysis. Afterwards, we calculated GT measures at the given threshold and used them in the K-means clustering algorithm. In this investigation, the density value arising from these calculations was 20% for the functional analysis, and 40% for the morphological analysis.

### Categorization of clinical variables

In order to better visualize cluster-wise comparisons with clinical variables, we categorized the latter. The number of ASMs taken by TLE participants was binarized: one group was composed of patients taking more than one ASM (n = 58) and the other composed of patients taking 1 or no ASMs (n = 39). The lifetime number of GTCS and age-at-first seizure were also binarized by dividing the group at the median. For the lifetime number of GTCS, 47 patients were in the group below the median (median of 4 lifetime GTCS) (4 subjects had unknown data), and for age-at-first-seizure, 50 patients were in the group below the median (median of age 20 years). Medication refractory (*yes* or *no*) and seizure lateralization (*left*, *right*, *bilateral*, *unknown*) are already categorical, therefore were not modified. Table 1 shows mean and SD for the beforementioned categorical clinical variables.

### K-means cluster analysis

Global GT metrics from TLE participants were scaled to a mean of zero and a standard deviation of one. Next, the optimal number of clusters for both functional and morphological connectivity was determined using the elbow method^15^ and silhouette method^16^. For both functional and morphological clusters the optimal cluster number was 2 using both the elbow method and average silhouette methods (Fig. 2S in Supplemental file). K-means clustering with K = 2 was performed with bootstrapping (1000 trials) to ensure stability of clustering. Final partitions were determined by the frequency of concurrence over the 1000 trials (“fpc”—Flexible Procedures for Clustering” 2.2‐3, Christian Henning R package). Clustering analysis was performed in R version 4.0.5.

## Supplementary Information

Supplementary Information.
