# Supplementary material for: Network phenotypes and their clinical significance in temporal lobe epilepsy using machine learning applications to morphological and functional graph theory metrics
Source: Sci Rep. 2022 Aug 24;12:14407. doi: 10.1038/s41598-022-18495-z (PMC9402557; doi:10.1038/s41598-022-18495-z)
Supplement: Supplementary file 1 — Supplementary Information. [file 41598_2022_18495_MOESM1_ESM.pdf]

Table 1S: Morphological Nodes

	Node	Abbrev.
1	lh_frontalpole	L_PoleF
2	lh_superiorfrontal	L_SupF
3	lh_rostralmiddlefrontal	L_RosMidF
4	lh_caudalmiddlefrontal	L_CaudMidF
5	lh_lateralorbitofrontal	L_LatOrbF
6	lh_medialorbitofrontal	L_MedOrbF
7	lh_parsopercularis	L_ParsOp
8	lh_parsorbitalis	L_ParsOrb
9	lh_parstriangularis	L_ParsTri
10	lh_precentral	L_Precent
11	lh_insula	L_Ins
12	lh_paracentral	L_Parac
13	lh_postcentral	L_Postc
14	lh_superiorparietal	L_SupP
15	lh_precuneus	L_Precun
16	lh_inferiorparietal	L_InfP
17	lh_supramarginal	L_Supram
18	lh_temporalpole	L_PoleT
19	lh_superiortemporal	L_SupT
20	lh_middletemporal	L_MidT
21	lh_inferiortemporal	L_InfT
22	lh_bankssstsT	L_Bankssts
23	lh_entorhinalT	L_Enthorh
24	lh_transversetemporal	L_TransvT
25	lh_parahippocampal	L_Parahipp
26	lh_fusiform	L_Fusif
27	lh_cuneus	L_Cuneus
28	lh_lateraloccipital	L_LatO
29	lh_lingual	L_Ling
30	lh_pericalcarine	L_Pericalc
31	lh_rostralanteriorcingulate	L_RosAntC
32	lh_caudalanteriorcingulate	L_CaudAntC
33	lh_isthmuscingulate	L_IsthmC
34	lh_posteriorcingulate	L_PostC
35	rh_frontalpole	R_PoleF
36	rh_superiorfrontal	R_SupF
37	rh_rostralmiddlefrontal	R_RosMidF
38	rh_caudalmiddlefrontal	R_CaudMidF
39	rh_lateralorbitofrontal	R_LatOrbF
40	rh_medialorbitofrontal	R_MedOrbF
41	rh_parsopercularis	R_ParsOp
42	rh_parsorbitalis	R_ParsOrb
43	rh_parstriangularis	R_ParsTri

44	rh_precentral	R_Precent
45	rh_insula	R_Ins
46	rh_paracentral	R_Parac
47	rh_postcentral	R_Postc
48	rh_superiorparietal	R_SupP
49	rh_precuneus	R_Precun
50	rh_inferiorparietal	R_InfP
51	rh_supramarginal	R_Supram
52	rh_temporalpole	R_PoleT
53	rh_superiortemporal	R_SupT
54	rh_middletemporal	R_MidT
55	rh_inferiortemporal	R_InfT
56	rh_bankssts	R_Bankssts
57	rh_entorhinal	R_Enthorh
58	rh_transversetemporal	R_TransvT
59	rh_parahippocampal	R_Parahipp
60	rh_fusiform	R_Fusif
61	rh_cuneus	R_Cuneus
62	rh_lateraloccipital	R_LatO
63	rh_lingual	R_Ling
64	rh_pericalcarine	R_Pericalc
65	rh_rostralanteriorcingulate	R_RosAntC
66	rh_caudalanteriorcingulate	R_CaudAntC
67	rh_isthmuscingulate	R_IsthmC
68	rh_posteriorcingulate	R_PostC
69	Left-Thalamus	L_Thal
70	Left-Caudate	L_Caud
71	Left-Putamen	L_Putam
72	Left-Pallidum	L_Pallid
73	Left-Hippocampus	L_Hippoc
74	Left-Amygdala	L_Amyg
75	Left-Accumbens-area	L_Accumb
76	Left-VentralDC	L_VentDC
77	Left-Cerebellum	L_Cereb
78	Right-Thalamus	R_Thal
79	Right-Caudate	R_Caud
80	Right-Putamen	R_Putam
81	Right-Pallidum	R_Pallid
82	Right-Hippocampus	R_Hippoc
83	Right-Amygdala	R_Amyg
84	Right-Accumbens-area	R_Accumb
85	Right-VentralDC	R_VentDC
86	Right-Cerebellum	R_Cereb
87	Brain-Stem	BrainStem

Table 2S: Functional Nodes

Glasser Parcel Number	Glasser Node Label	Glasser Region (22 total)
1	L_Primary Visual Cortex	Primary Visual Cortex (V1)
2	L_Medial Superior Temporal Area	MT+ Complex and Neighbors
3	L_Sixth Visual Area	Dorsal Stream
4	L_Second Visual Area	Early Visual Cortex
5	L_Third Visual Area	Early Visual Cortex
6	L_Fourth Visual Area	Early Visual Cortex
7	L_Eighth Visual Area	Ventral Stream
8	L_Primary Motor Cortex	Somatosensory and Motor Cortex
9	L_Primary Sensory Cortex	Somatosensory and Motor Cortex
10	L_Frontal Eye Fields	Premotor Cortex
11	L_Premotor Eye Field	Premotor Cortex
12	L_Area 55b	Premotor Cortex
13	L_Area V3A	Dorsal Stream
14	L_RetroSplenial Complex	Posterior Cingulate Cortex
15	L_Parieto-Occipital Sulcus Area 2	Posterior Cingulate Cortex
16	L_Seventh Visual Area	Dorsal Stream
17	L_IntraParietal Sulcus Area 1	Dorsal Stream
18	L_Fusiform Face Complex	Ventral Stream
19	L_Area V3B	Dorsal Stream
20	L_Area Lateral Occipital 1	MT+ Complex and Neighbors
21	L_Area Lateral Occipital 2	MT+ Complex and Neighbors
22	L_Posterior InferoTemporalComplex	Ventral Stream
23	L_Middle Temporal Area	MT+ Complex and Neighbors
24	L_Primary Auditory Cortex	Early Auditory Cortex
25	L_PeriSylvian Language Area	Temporal-Parietal-Occipital Junction
26	L_Superior Frontal Language Area	Dorsolateral Prefrontal Cortex
27	L_PreCuneus Visual Area	Posterior Cingulate Cortex
28	L_Superior Temporal Visual Area	Temporal-Parietal-Occipital Junction
29	L_Medial Area 7P	Superior Parietal and IPS Cortex
30	L_Area 7m	Posterior Cingulate Cortex
31	L_Parieto-Occipital Sulcus Area 1	Posterior Cingulate Cortex
32	L_Area 23d	Posterior Cingulate Cortex
33	L_Area ventral 23 a+b	Posterior Cingulate Cortex
34	L_Area dorsal 23 a+b	Posterior Cingulate Cortex
35	L_Area 31p ventral	Posterior Cingulate Cortex
36	L_Area 5m	Sensorimotor Associated Paracentral Lobular and Mid Cingulate Cortex

37	L_Area 5m ventral	Sensorimotor Associated Paracentral Lobular and Mid Cingulate Cortex
38	L_Area 23c	Posterior Cingulate Cortex
39	L_Area 5L	Sensorimotor Associated Paracentral Lobular and Mid Cingulate Cortex
40	L_Dorsal Area 24d	Sensorimotor Associated Paracentral Lobular and Mid Cingulate Cortex
41	L_Ventral Area 24d	Sensorimotor Associated Paracentral Lobular and Mid Cingulate Cortex
42	L_Lateral Area 7A	Superior Parietal and IPS Cortex
43	L_Supplementary and Cingulate Eye Field	Sensorimotor Associated Paracentral Lobular and Mid Cingulate Cortex
44	L_Area 6m anterior	Sensorimotor Associated Paracentral Lobular and Mid Cingulate Cortex
45	L_Medial Area 7A	Superior Parietal and IPS Cortex
46	L_Lateral Area 7P	Superior Parietal and IPS Cortex
47	L_Area 7PC	Superior Parietal and IPS Cortex
48	L_Area Lateral IntraParietal ventral	Superior Parietal and IPS Cortex
49	L_Ventral IntraParietal Complex	Superior Parietal and IPS Cortex
50	L_Medial IntraParietal Area	Superior Parietal and IPS Cortex
51	L_Area 1	Somatosensory and Motor Cortex
52	L_Area 2	Somatosensory and Motor Cortex
53	L_Area 3a	Somatosensory and Motor Cortex
54	L_Dorsal area 6	Premotor Cortex
55	L_Area 6mp	Sensorimotor Associated Paracentral Lobular and Mid Cingulate Cortex
56	L_Ventral Area 6	Premotor Cortex
57	L_Area Posterior 24 prime	Anterior Cingulate and Medial Prefrontal Cortex
58	L_Area 33 prime	Anterior Cingulate and Medial Prefrontal Cortex
59	L_Anterior 24 prime	Anterior Cingulate and Medial Prefrontal Cortex
60	L_Area p32 prime	Anterior Cingulate and Medial Prefrontal Cortex
61	L_Area a24	Anterior Cingulate and Medial Prefrontal Cortex
62	L_Area dorsal 32	Anterior Cingulate and Medial Prefrontal Cortex
63	L_Area 8BM	Anterior Cingulate and Medial Prefrontal Cortex

64	L_Area p32	Anterior Cingulate and Medial Prefrontal Cortex
65	L_Area 10r	Anterior Cingulate and Medial Prefrontal Cortex
66	L_Area 47m	Orbital and Polar Frontal Cortex
67	L_Area 8Av	Dorsolateral Prefrontal Cortex
68	L_Area 8Ad	Dorsolateral Prefrontal Cortex
69	L_Area 9 Middle	Anterior Cingulate and Medial Prefrontal Cortex
70	L_Area 8B Lateral	Dorsolateral Prefrontal Cortex
71	L_Area 9 Posterior	Dorsolateral Prefrontal Cortex
72	L_Area 10d	Orbital and Polar Frontal Cortex
73	L_Area 8C	Dorsolateral Prefrontal Cortex
74	L_Area 44	Inferior Frontal Cortex
75	L_Area 45	Inferior Frontal Cortex
76	L_Area 47l (47 lateral)	Inferior Frontal Cortex
77	L_Area anterior 47r	Orbital and Polar Frontal Cortex
78	L_Rostral Area 6	Premotor Cortex
79	L_Area IFJa	Inferior Frontal Cortex
80	L_Area IFJp	Inferior Frontal Cortex
81	L_Area IFSp	Inferior Frontal Cortex
82	L_Area IFSa	Inferior Frontal Cortex
83	L_Area posterior 9-46v	Dorsolateral Prefrontal Cortex
84	L_Area 46	Dorsolateral Prefrontal Cortex
85	L_Area anterior 9-46v	Dorsolateral Prefrontal Cortex
86	L_Area 9-46d	Dorsolateral Prefrontal Cortex
87	L_Area 9 anterior	Dorsolateral Prefrontal Cortex
88	L_Area 10v	Anterior Cingulate and Medial Prefrontal Cortex
89	L_Area anterior 10p	Orbital and Polar Frontal Cortex
90	L_Polar 10p	Orbital and Polar Frontal Cortex
91	L_Area 11l	Orbital and Polar Frontal Cortex
92	L_Area 13l	Orbital and Polar Frontal Cortex
93	L_Orbital Frontal Complex	Orbital and Polar Frontal Cortex
94	L_Area 47s	Orbital and Polar Frontal Cortex
95	L_Area Lateral IntraParietal dorsal	Superior Parietal and IPS Cortex
96	L_Area 6 anterior	Premotor Cortex
97	L_Inferior 6-8 Transitional Area	Dorsolateral Prefrontal Cortex
98	L_Superior 6-8 Translational Area	Dorsolateral Prefrontal Cortex
99	L_Area 43	Posterior Opercular Cortex

100	L_Area OP4/PV	Posterior Opercular Cortex
101	L_Area OP1/SII	Posterior Opercular Cortex
102	L_Area OP2-3/VS	Posterior Opercular Cortex
103	L_Area 52	Insular and Frontal Opercular Cortex
104	L_RetroInsular Cortex	Early Auditory Cortex
105	L_Area PFcm	Posterior Opercular Cortex
106	L_Posterior Insular Area 2	Insular and Frontal Opercular Cortex
107	L_Area TA2	Auditory Association Cortex
108	L_Frontal OPercular Area 4	Insular and Frontal Opercular Cortex
109	L_Middle Insular Area	Insular and Frontal Opercular Cortex
110	L_Pirform Cortex	Insular and Frontal Opercular Cortex
111	L_Anterior Ventral Insular Area	Insular and Frontal Opercular Cortex
112	L_Anterior Agranular Insula Complex	Insular and Frontal Opercular Cortex
113	L_Frontal OPercular Area 1	Posterior Opercular Cortex
114	L_Frontal OPercular Area 3	Insular and Frontal Opercular Cortex
115	L_Frontal OPercular Area 2	Insular and Frontal Opercular Cortex
116	L_Area PFt	Inferior Parietal Cortex
117	L_Anterior IntraParietal Area	Superior Parietal and IPS Cortex
118	L_Entorhinal Cortex	Medial Temporal Cortex
119	L_PreSubiculum	Medial Temporal Cortex
120	L_Hippocampus	Medial Temporal Cortex
121	L_ProStriate Area	Posterior Cingulate Cortex
122	L_Perirhinal Ectorhinal Cortex	Medial Temporal Cortex
123	L_Area STGa	Auditory Association Cortex
124	L_ParaBelt Complex	Early Auditory Cortex
125	L_Auditory 5 Complex	Auditory Association Cortex
126	L_ParaHippocampal Area 1	Medial Temporal Cortex
127	L_ParaHippocampal Area 3	Medial Temporal Cortex
128	L_Area STSd anterior	Auditory Association Cortex
129	L_Area STSd posterior	Auditory Association Cortex
130	L_Area STSv posterior	Auditory Association Cortex
131	L_Area TG dorsal	Lateral Temporal Cortex
132	L_Area TE1 anterior	Lateral Temporal Cortex

133	L_Area TE1 posterior	Lateral Temporal Cortex
134	L_Area TE2 anterior	Lateral Temporal Cortex
135	L_Area TF	Lateral Temporal Cortex
136	L_Area TE2 posterior	Lateral Temporal Cortex
137	L_Area PHT	Lateral Temporal Cortex
138	L_Area PH	MT+ Complex and Neighbors
139	L_Area TemporoParietoOccipital Junction 1	Temporal-Parietal-Occipital Junction
140	L_Area TemporoParietoOccipital Junction 2	Temporal-Parietal-Occipital Junction
141	L_Area TemporoParietoOccipital Junction 3	Temporal-Parietal-Occipital Junction
142	L_Dorsal Transitional Visual Area	Posterior Cingulate Cortex
143	L_Area PGp	Inferior Parietal Cortex
144	L_Area IntraParietal 2	Inferior Parietal Cortex
145	L_Area IntraParietal 1	Inferior Parietal Cortex
146	L_Area IntraParietal 0	Inferior Parietal Cortex
147	L_Area PF opercular	Inferior Parietal Cortex
148	L_Area PF Complex	Inferior Parietal Cortex
149	L_Area PFm Complex	Inferior Parietal Cortex
150	L_Area PGi	Inferior Parietal Cortex
151	L_Area PGs	Inferior Parietal Cortex
152	L_Area V6A	Dorsal Stream
153	L_VentroMedial Visual Area 1	Ventral Stream
154	L_VentroMedial Visual Area 3	Ventral Stream
155	L_ParaHippocampal Area 2	Medial Temporal Cortex
156	L_Area V4t	MT+ Complex and Neighbors
157	L_Area FST	MT+ Complex and Neighbors
158	L_Area V3CD	MT+ Complex and Neighbors
159	L_Area Lateral Occipital 3	MT+ Complex and Neighbors
160	L_VentroMedial Visual Area 2	Ventral Stream
161	L_Area 31pd	Posterior Cingulate Cortex
162	L_Area 31a	Posterior Cingulate Cortex

163	L_Ventral Visual Complex	Ventral Stream
164	L_Area 25	Anterior Cingulate and Medial Prefrontal Cortex
165	L_Area s32	Anterior Cingulate and Medial Prefrontal Cortex
166	L_posterior OFC Complex	Orbital and Polar Frontal Cortex
167	L_Area Posterior Insular 1	Insular and Frontal Opercular Cortex
168	L_Insular Granular Complex	Insular and Frontal Opercular Cortex
169	L_Area Frontal Opercular 5	Insular and Frontal Opercular Cortex
170	L_Area posterior 10p	Orbital and Polar Frontal Cortex
171	L_Area posterior 47r	Inferior Frontal Cortex
172	L_Area TG Ventral	Lateral Temporal Cortex
173	L_Medial Belt Complex	Early Auditory Cortex
174	L_Lateral Belt Complex	Early Auditory Cortex
175	L_Auditory 4 Complex	Auditory Association Cortex
176	L_Area STSv anterior	Auditory Association Cortex
177	L_Area TE1 Middle	Lateral Temporal Cortex
178	L_Para-Insular Area	Insular and Frontal Opercular Cortex
179	L_Area anterior 32 prime	Anterior Cingulate and Medial Prefrontal Cortex
180	L_Area posterior 24	Anterior Cingulate and Medial Prefrontal Cortex
181	R_Primary Visual Cortex	Primary Visual Cortex (V1)
182	R_Medial Superior Temporal Area	MT+ Complex and Neighbors
183	R_Sixth Visual Area	Dorsal Stream
184	R_Second Visual Area	Early Visual Cortex
185	R_Third Visual Area	Early Visual Cortex
186	R_Fourth Visual Area	Early Visual Cortex
187	R_Eighth Visual Area	Ventral Stream
188	R_Primary Motor Cortex	Somatosensory and Motor Cortex
189	R_Primary Sensory Cortex	Somatosensory and Motor Cortex
190	R_Frontal Eye Fields	Premotor Cortex
191	R_Premotor Eye Field	Premotor Cortex
192	R_Area 55b	Premotor Cortex
193	R_Area V3A	Dorsal Stream
194	R_RetroSplenial Complex	Posterior Cingulate Cortex
195	R_Parieto-Occipital Sulcus Area 2	Posterior Cingulate Cortex

196	R_Seventh Visual Area	Dorsal Stream
197	R_IntraParietal Sulcus Area 1	Dorsal Stream
198	R_Fusiform Face Complex	Ventral Stream
199	R_Area V3B	Dorsal Stream
200	R_Area Lateral Occipital 1	MT+ Complex and Neighbors
201	R_Area Lateral Occipital 2	MT+ Complex and Neighbors
202	R_Posterior InferoTemporalComplex	Ventral Stream
203	R_Middle Temporal Area	MT+ Complex and Neighbors
204	R_Primary Auditory Cortex	Early Auditory Cortex
205	R_PeriSylvian Language Area	Temporal-Parietal-Occipital Junction
206	R_Superior Frontal Language Area	Dorsolateral Prefrontal Cortex
207	R_PreCuneus Visual Area	Posterior Cingulate Cortex
208	R_Superior Temporal Visual Area	Temporal-Parietal-Occipital Junction
209	R_Medial Area 7P	Superior Parietal and IPS Cortex
210	R_Area 7m	Posterior Cingulate Cortex
211	R_Parieto-Occipital Sulcus Area 1	Posterior Cingulate Cortex
212	R_Area 23d	Posterior Cingulate Cortex
213	R_Area ventral 23 a+b	Posterior Cingulate Cortex
214	R_Area dorsal 23 a+b	Posterior Cingulate Cortex
215	R_Area 31p ventral	Posterior Cingulate Cortex
216	R_Area 5m	Sensorimotor Associated Paracentral Lobular and Mid Cingulate Cortex
217	R_Area 5m ventral	Sensorimotor Associated Paracentral Lobular and Mid Cingulate Cortex
218	R_Area 23c	Posterior Cingulate Cortex
219	R_Area 5L	Sensorimotor Associated Paracentral Lobular and Mid Cingulate Cortex
220	R_Dorsal Area 24d	Sensorimotor Associated Paracentral Lobular and Mid Cingulate Cortex
221	R_Ventral Area 24d	Sensorimotor Associated Paracentral Lobular and Mid Cingulate Cortex
222	R_Lateral Area 7A	Superior Parietal and IPS Cortex
223	R_Supplementary and Cingulate Eye Field	Sensorimotor Associated Paracentral Lobular and Mid Cingulate Cortex
224	R_Area 6m anterior	Sensorimotor Associated Paracentral Lobular and Mid Cingulate Cortex
225	R_Medial Area 7A	Superior Parietal and IPS Cortex
226	R_Lateral Area 7P	Superior Parietal and IPS Cortex
227	R_Area 7PC	Superior Parietal and IPS Cortex
228	R_Area Lateral IntraParietal ventral	Superior Parietal and IPS Cortex
229	R_Ventral IntraParietal Complex	Superior Parietal and IPS Cortex
230	R_Medial IntraParietal Area	Superior Parietal and IPS Cortex

231	R_Area 1	Somatosensory and Motor Cortex
232	R_Area 2	Somatosensory and Motor Cortex
233	R_Area 3a	Somatosensory and Motor Cortex
234	R_Dorsal area 6	Premotor Cortex
235	R_Area 6mp	Sensorimotor Associated Paracentral Lobular and Mid Cingulate Cortex
236	R_Ventral Area 6	Premotor Cortex
237	R_Area Posterior 24 prime	Anterior Cingulate and Medial Prefrontal Cortex
238	R_Area 33 prime	Anterior Cingulate and Medial Prefrontal Cortex
239	R_Anterior 24 prime	Anterior Cingulate and Medial Prefrontal Cortex
240	R_Area p32 prime	Anterior Cingulate and Medial Prefrontal Cortex
241	R_Area a24	Anterior Cingulate and Medial Prefrontal Cortex
242	R_Area dorsal 32	Anterior Cingulate and Medial Prefrontal Cortex
243	R_Area 8BM	Anterior Cingulate and Medial Prefrontal Cortex
244	R_Area p32	Anterior Cingulate and Medial Prefrontal Cortex
245	R_Area 10r	Anterior Cingulate and Medial Prefrontal Cortex
246	R_Area 47m	Orbital and Polar Frontal Cortex
247	R_Area 8Av	Dorsolateral Prefrontal Cortex
248	R_Area 8Ad	Dorsolateral Prefrontal Cortex
249	R_Area 9 Middle	Anterior Cingulate and Medial Prefrontal Cortex
250	R_Area 8B Lateral	Dorsolateral Prefrontal Cortex
251	R_Area 9 Posterior	Dorsolateral Prefrontal Cortex
252	R_Area 10d	Orbital and Polar Frontal Cortex
253	R_Area 8C	Dorsolateral Prefrontal Cortex
254	R_Area 44	Inferior Frontal Cortex
255	R_Area 45	Inferior Frontal Cortex
256	R_Area 47l (47 lateral)	Inferior Frontal Cortex
257	R_Area anterior 47r	Orbital and Polar Frontal Cortex
258	R_Rostral Area 6	Premotor Cortex
259	R_Area IFJa	Inferior Frontal Cortex
260	R_Area IFJp	Inferior Frontal Cortex
261	R_Area IFSp	Inferior Frontal Cortex

262	R_Area IFSa	Inferior Frontal Cortex
263	R_Area posterior 9-46v	Dorsolateral Prefrontal Cortex
264	R_Area 46	Dorsolateral Prefrontal Cortex
265	R_Area anterior 9-46v	Dorsolateral Prefrontal Cortex
266	R_Area 9-46d	Dorsolateral Prefrontal Cortex
267	R_Area 9 anterior	Dorsolateral Prefrontal Cortex
268	R_Area 10v	Anterior Cingulate and Medial Prefrontal Cortex
269	R_Area anterior 10p	Orbital and Polar Frontal Cortex
270	R_Polar 10p	Orbital and Polar Frontal Cortex
271	R_Area 11l	Orbital and Polar Frontal Cortex
272	R_Area 13l	Orbital and Polar Frontal Cortex
273	R_Orbital Frontal Complex	Orbital and Polar Frontal Cortex
274	R_Area 47s	Orbital and Polar Frontal Cortex
275	R_Area Lateral IntraParietal dorsal	Superior Parietal and IPS Cortex
276	R_Area 6 anterior	Premotor Cortex
277	R_Inferior 6-8 Transitional Area	Dorsolateral Prefrontal Cortex
278	R_Superior 6-8 Translational Area	Dorsolateral Prefrontal Cortex
279	R_Area 43	Posterior Opercular Cortex
280	R_Area OP4/PV	Posterior Opercular Cortex
281	R_Area OP1/SII	Posterior Opercular Cortex
282	R_Area OP2-3/VS	Posterior Opercular Cortex
283	R_Area 52	Insular and Frontal Opercular Cortex
284	R_RetroInsular Cortex	Early Auditory Cortex
285	R_Area PFcm	Posterior Opercular Cortex
286	R_Posterior Insular Area 2	Insular and Frontal Opercular Cortex
287	R_Area TA2	Auditory Association Cortex
288	R_Frontal OPercular Area 4	Insular and Frontal Opercular Cortex
289	R_Middle Insular Area	Insular and Frontal Opercular Cortex
290	R_Pirform Cortex	Insular and Frontal Opercular Cortex
291	R_Anterior Ventral Insular Area	Insular and Frontal Opercular Cortex
292	R_Anterior Agranular Insula Complex	Insular and Frontal Opercular Cortex
293	R_Frontal OPercular Area 1	Posterior Opercular Cortex
294	R_Frontal OPercular Area 3	Insular and Frontal Opercular Cortex

295	R_Frontal OPercular Area 2	Insular and Frontal Opercular Cortex
296	R_Area PFt	Inferior Parietal Cortex
297	R_Anterior IntraParietal Area	Superior Parietal and IPS Cortex
298	R_Entorhinal Cortex	Medial Temporal Cortex
299	R_PreSubiculum	Medial Temporal Cortex
300	R_Hippocampus	Medial Temporal Cortex
301	R_ProStriate Area	Posterior Cingulate Cortex
302	R_Perirhinal Ectorhinal Cortex	Medial Temporal Cortex
303	R_Area STGa	Auditory Association Cortex
304	R_ParaBelt Complex	Early Auditory Cortex
305	R_Auditory 5 Complex	Auditory Association Cortex
306	R_ParaHippocampal Area 1	Medial Temporal Cortex
307	R_ParaHippocampal Area 3	Medial Temporal Cortex
308	R_Area STSd anterior	Auditory Association Cortex
309	R_Area STSd posterior	Auditory Association Cortex
310	R_Area STSv posterior	Auditory Association Cortex
311	R_Area TG dorsal	Lateral Temporal Cortex
312	R_Area TE1 anterior	Lateral Temporal Cortex
313	R_Area TE1 posterior	Lateral Temporal Cortex
314	R_Area TE2 anterior	Lateral Temporal Cortex
315	R_Area TF	Lateral Temporal Cortex
316	R_Area TE2 posterior	Lateral Temporal Cortex
317	R_Area PHT	Lateral Temporal Cortex
318	R_Area PH	MT+ Complex and Neighbors
319	R_Area TemporoParietoOccipital Junction 1	Temporal-Parietal-Occipital Junction
320	R_Area TemporoParietoOccipital Junction 2	Temporal-Parietal-Occipital Junction
321	R_Area TemporoParietoOccipital Junction 3	Temporal-Parietal-Occipital Junction
322	R_Dorsal Transitional Visual Area	Posterior Cingulate Cortex
323	R_Area PGp	Inferior Parietal Cortex
324	R_Area IntraParietal 2	Inferior Parietal Cortex
325	R_Area IntraParietal 1	Inferior Parietal Cortex

326	R_Area IntraParietal 0	Inferior Parietal Cortex
327	R_Area PF opercular	Inferior Parietal Cortex
328	R_Area PF Complex	Inferior Parietal Cortex
329	R_Area PFm Complex	Inferior Parietal Cortex
330	R_Area PGi	Inferior Parietal Cortex
331	R_Area PGs	Inferior Parietal Cortex
332	R_Area V6A	Dorsal Stream
333	R_VentroMedial Visual Area 1	Ventral Stream
334	R_VentroMedial Visual Area 3	Ventral Stream
335	R_ParaHippocampal Area 2	Medial Temporal Cortex
336	R_Area V4t	MT+ Complex and Neighbors
337	R_Area FST	MT+ Complex and Neighbors
338	R_Area V3CD	MT+ Complex and Neighbors
339	R_Area Lateral Occipital 3	MT+ Complex and Neighbors
340	R_VentroMedial Visual Area 2	Ventral Stream
341	R_Area 31pd	Posterior Cingulate Cortex
342	R_Area 31a	Posterior Cingulate Cortex
343	R_Ventral Visual Complex	Ventral Stream
344	R_Area 25	Anterior Cingulate and Medial Prefrontal Cortex
345	R_Area s32	Anterior Cingulate and Medial Prefrontal Cortex
346	R_posterior OFC Complex	Orbital and Polar Frontal Cortex
347	R_Area Posterior Insular 1	Insular and Frontal Opercular Cortex
348	R_Insular Granular Complex	Insular and Frontal Opercular Cortex
349	R_Area Frontal Opercular 5	Insular and Frontal Opercular Cortex
350	R_Area posterior 10p	Orbital and Polar Frontal Cortex
351	R_Area posterior 47r	Inferior Frontal Cortex
352	R_Area TG Ventral	Lateral Temporal Cortex
353	R_Medial Belt Complex	Early Auditory Cortex
354	R_Lateral Belt Complex	Early Auditory Cortex
355	R_Auditory 4 Complex	Auditory Association Cortex

356	R_Area STSv anterior	Auditory Association Cortex
357	R_Area TE1 Middle	Lateral Temporal Cortex
358	R_Para-Insular Area	Insular and Frontal Opercular Cortex
359	R_Area anterior 32 prime	Anterior Cingulate and Medial Prefrontal Cortex
360	R_Area posterior 24	Anterior Cingulate and Medial Prefrontal Cortex
361	L_Amygdala	Subcortical
362	R_Amygdala	Subcortical
363	L_Hippocampus	Subcortical
364	R_Hippocampus	Subcortical
365	L_Accumbens	Subcortical
366	R_Accumbens	Subcortical
367	L_Caudate	Subcortical
368	R_Caudate	Subcortical
369	L_Pallidum	Subcortical
370	R_Pallidum	Subcortical
371	L_Putamen	Subcortical
372	R_Putamen	Subcortical
373	L_Thalamus	Subcortical
374	R_Thalamus	Subcortical
375	BrainStem	Subcortical
376	L_VentralDiencephalon	Subcortical
377	R_VentralDiencephalon	Subcortical
378	L_Cerebellum	Subcortical
379	R_Cerebellum	Subcortical

Supplemental Figures

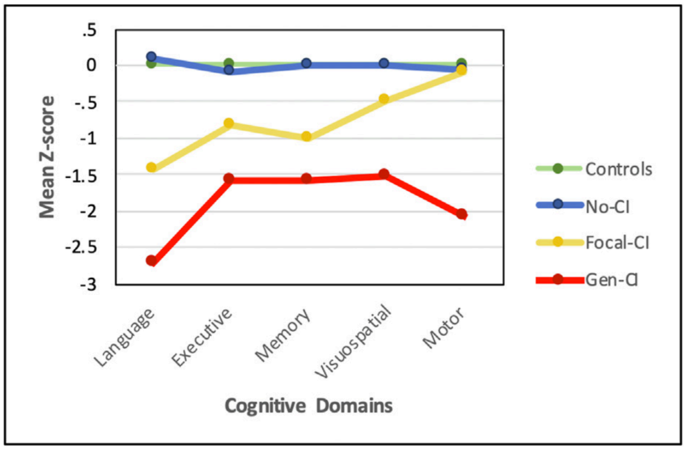

Figure 1S. Cognitive phenotype groups from the Epilepsy Connectome Project. Controls (green, *N* = 30), No Cognitive Impairment (No-CI) being the most intact (blue, *N* = 57), Focal Cognitive Impairment (Focal-CI) (yellow, *N* = 34) with leading impairments in language, executive function and memory; and Generalized Cognitive Impairment (Gen-CI) (red, *N* = 20) being the most impaired overall. Plotted are mean domain scores for each phenotype. Reprinted from Hermann et al. (2020).

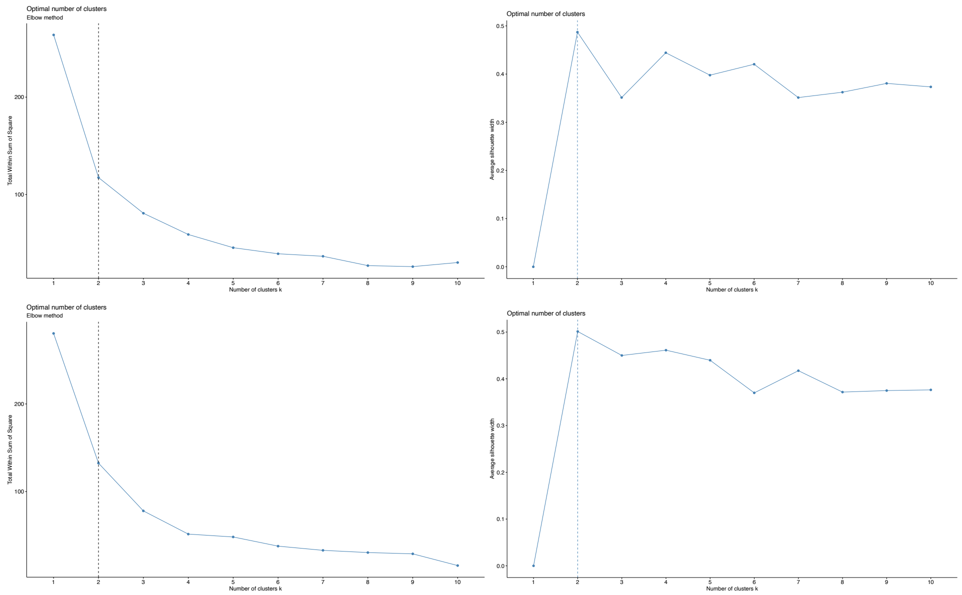

Figure 2S. (Left) Elbow method and (right) average silhouette method displaying 2 as the optimal number of clusters for both (top) functional and (bottom) morphological GT measures.

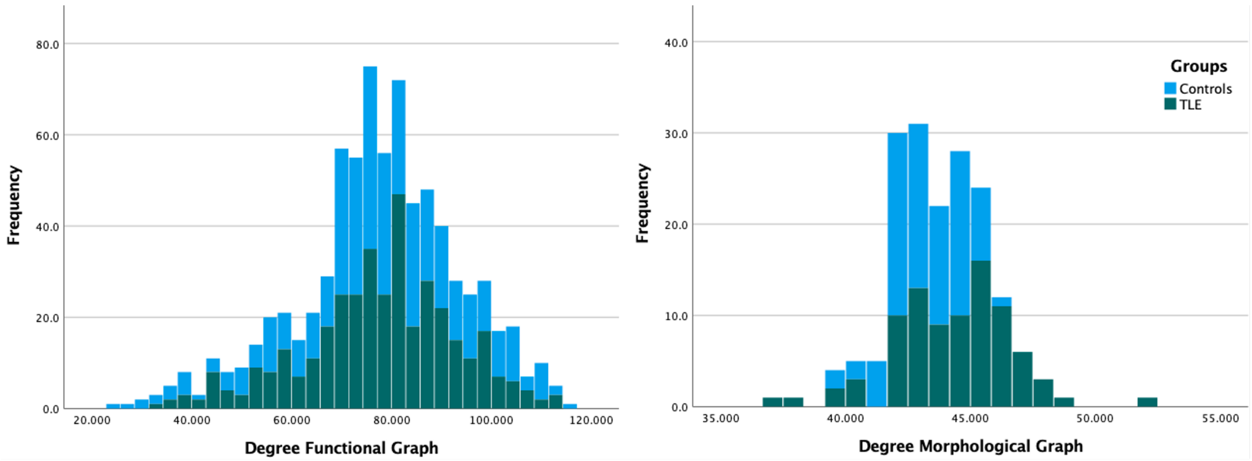

Figure 3S. (Left) Histogram of the degree distribution for the functional graph, and (right) the morphological graph in controls (blue) and TLE (green).
